# Preoperative risk factors for postoperative blood transfusion after hip fracture surgery: establishment of a nomogram

**DOI:** 10.1186/s13018-021-02557-5

**Published:** 2021-06-23

**Authors:** Fu Cheng Bian, Xiao Kang Cheng, Yong Sheng An

**Affiliations:** 1 Chengde Medical University, Chengde, 067000 Hebei China; 2 Department of Minimally Invasive Spine Surgery, Chengde Medical University Affiliated Hospital, Chengde, 067000 Hebei China

**Keywords:** Blood transfusion, Hip fracture, Risk factor, Nomogram

## Abstract

**Background:**

This study aimed to explore the preoperative risk factors related to blood transfusion after hip fracture operations and to establish a nomogram prediction model. The application of this model will likely reduce unnecessary transfusions and avoid wasting blood products.

**Methods:**

This was a retrospective analysis of all patients undergoing hip fracture surgery from January 2013 to January 2020. Univariate and multivariate logistic regression analyses were used to evaluate the association between preoperative risk factors and blood transfusion after hip fracture operations. Finally, the risk factors obtained from the multivariate regression analysis were used to establish the nomogram model. The validation of the nomogram was assessed by the concordance index (C-index), the receiver operating characteristic (ROC) curve, decision curve analysis (DCA), and calibration curves.

**Results:**

A total of 820 patients were included in the present study for evaluation. Multivariate logistic regression analysis demonstrated that low preoperative hemoglobin (Hb), general anesthesia (GA), non-use of tranexamic acid (TXA), and older age were independent risk factors for blood transfusion after hip fracture operation. The C-index of this model was 0.86 (95% CI, 0.83–0.89). Internal validation proved the nomogram model’s adequacy and accuracy, and the results showed that the predicted value agreed well with the actual values.

**Conclusions:**

A nomogram model was developed based on independent risk factors for blood transfusion after hip fracture surgery. Preoperative intervention can effectively reduce the incidence of blood transfusion after hip fracture operations.

## Background

The incidence of hip fracture is increasing yearly, and research has also been conducted worldwide [1]. However, bone mineral density is decreased in elderly individuals, and low-energy trauma can lead to hip fracture. Cooper et al. [2] illustrated that by 2050, there would be 6.26 million incident hip fractures worldwide. The disability and death rates of hip fractures are high. Patients with a hip fracture will suffer a loss of independent living ability, which has caused a significant burden to family members and society [3].

Elderly patients who undergo surgical treatment have higher odds of receiving blood transfusion, and the probability of blood transfusion after hip fracture surgery can be as high as 56% [4]. There are many complications after hip fracture, such as low hemoglobin and albumin levels [5]. Blood transfusion increases the risk of joint infection around the incision and surgical area while potentially increasing the risk of infectious diseases, hemolysis, and immune reactions [6]. Therefore, early intervention before surgery based on patients’ preoperative status and laboratory indicators can greatly reduce the risk of postoperative blood transfusion, and avoid the waste of blood products [7]. Hemostatic polymeric materials or hemostatic agents have been used in clinical practice for a long time, but there are drawbacks to the current clinical treatment with these materials [8]. Recently, Wang et al. [9] established a model by investigating the influencing factors of postoperative blood transfusion in femoral neck fracture and the obtained independent risk factors associated with postoperative blood transfusion. However, this study involved a single fracture type. The number of cases was relatively small, so the possibility of offset existed because the prediction model’s construction lacked considerable sample support.

Unlike previously reported studies, the present investigation included a large sample size, focusing on collecting preoperative risk factors for hip fracture patients. Independent risk factors related to postoperative blood transfusion were obtained using regression analysis, and the nomogram model was established. Through the scientific and systematic evaluation of the probability of postoperative blood transfusion, the nomogram model’s application before surgery can more accurately guide surgeons to intervene and treat patients in advance, reduce the occurrence of postoperative blood transfusion.

## Methods

### Patients

In total, 862 patients who underwent surgery for hip fracture between January 2013 and January 2020 were enrolled, and the ethics committee approved the trial at our hospital. The inclusion criteria were as follows: (1) low-energy injury (e.g., osteoporotic fracture); (2) unilateral fracture without other severe injuries; and (3) no history of antiplatelet drugs, non-steroidal anti-inflammatory, or vasoactive drug application within 1 week preoperatively. The exclusion criteria were as follows: (1) multiple, high-energy injuries; (2) apparent bone destruction, pathological fracture, or severe hematologic disorders; (3) open fracture with increased risk of infection; (4) American Society of Anesthesiologists scores greater than grade III. Of these, 42 patients were excluded: 26 developed severe delirium symptoms or severe procedure-related complications postoperatively (such as dislocation and deep infection), and 16 were transferred to the intensive care unit for continued treatment.

Ultimately, 820 patients were included in the study, and they were randomly divided into a training set (70%) and a testing set (30%). Patients in the training set were used to develop the nomogram model, whereas patients in the testing set were used to validate the resulting nomogram. The use of TXA involved a single dose of intravenous TXA (15 mg/kg) 30 min prior to surgery in the study [10]. According to the World Health Organization criteria, the degree of anemia was classified as mild (male, 11-12.9 g/dl; female, 11-11.9 g/dl), moderate (8-10.9 g/dl), or severe (< 8 g/dl) [11]. The transfusion of blood products should occur through different transfusion strategy according to the patient’s actual situation rather than merely using a certain transfusion strategy [12]. The standard of blood transfusion used in our hospital was that severe or moderate patients with heart rate > 100 bpm, systolic blood pressure < 90 mmHg, depressed mood, or extreme weakness would be considered for blood transfusion.

### Data collection

In this study, the preoperative factors affecting postoperative blood transfusion were collected through the electronic medical record system. All preoperative factors were taken from previous studies and included age, sex, BMI, preoperative hemoglobin (Hb), TXA (used or not used), type of anesthesia (GA or combined spinal-epidural anesthesia [CSEA]), preoperative waiting time, type of operation (hip arthroplasty, internal fixation), and type of fracture (femoral neck fracture or inter-trochanteric fracture). In addition, we reviewed the previous history of the patients, including diabetes, hypertension, smoking (> 10 years), and drinking of alcohol (> 10 years).

### Statistical analysis

Data were analyzed using the SPSS 26 software for Windows (IBM Corp., Armonk, NY, USA). First, the risk factors that may affect postoperative blood transfusion were classified. Student’s t test or the Mann-Whitney U test was used to perform transfusion and non-transfusion group comparisons for quantitative variables. Categorical variables were compared using the chi-square or Fisher’s exact test. Second, all the training set factors were included in the univariate and multivariate logistic regression analyses to exclude unrelated risk factors. The independent risk factors obtained based on the multivariate regression method were used to construct a nomogram model with the “rms” package of R software (version 3.6.1).

Finally, the C-index, the area under the ROC curve (AUC), calibration curve, and DCA were used to evaluate the predictive ability and performance of the risk model. The C-index enables evaluation of the predictive accuracy and discriminative ability of nomograms [13]. The C-index values ranged from 0.5-1.0, with low accuracy (< 0.5), moderate accuracy (0.5- 0.7), high accuracy (0.7 -0.9), and extreme accuracy (> 0.9). A calibration curve was used to compare the actual risk and predicted risk. The clinical usefulness of the nomogram was estimated by DCA based on the net benefit and threshold probabilities. Statistical tests used *p <*0.05 as a significance level.

## Results

**Fig. 1:**
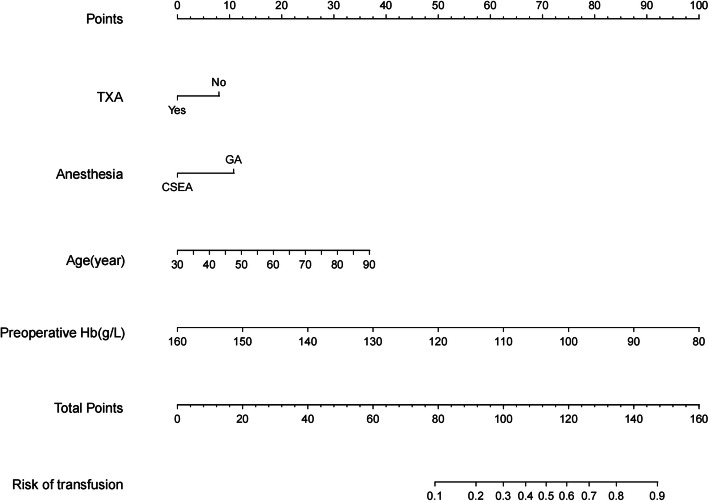
Nomogram for predicting postoperative blood transfusion in patients with hip fracture

A total of 820 patients were included in this study: 576 patients were included in the training set, and 244 patients were included in the testing set. In the training set, the transfusion group was compared with the non-transfusion group, and the results showed that age, sex, BMI, preoperative Hb, TXA, diabetes, and smoking were correlated with postoperative blood transfusion. However, there was no correlation between the type of anesthesia and postoperative blood transfusion (Table 1). Multivariate analysis showed that the type of anesthesia was an independent risk factor for blood transfusion after hip fracture surgery, so this factor was included in the final modeling. Risk factors (low preoperative Hb, GA, non-use of TXA, older age) for postoperative blood transfusion were obtained by multivariate regression in the training set (Table 2). A new nomogram was constructed to evaluate the postoperative blood transfusion probability after hip fracture (Fig. 1).

**Table 1:** Preoperative demographic characteristics in the training set

Characteristics	Transfusion (n = 111)	Non-transfusion (n = 465)	t/z/χ^**2**^	***P***
Age (year)	77 (69-82)	66 (56-77)	7.87	< 0.01*
Sex			7.49	< 0.01*
Female	83 (22.7%)	283 (77.3%)		
Male	28 (13.3%)	182 (86.7%)		
BMI (kg/m^2^)	22 (20.3-24)	23.2 (20.8-25.4)	3.16	< 0.01*
Hb (g/L)	105.96 ± 14.08	126.62 ± 15.18	11.07	< 0.01*
TXA			13.29	< 0.01*
No	79 (24.6%)	242 (75.4%)		
Yes	32 (12.5%)	223 (87.5%)		
Type of anesthesia			1.33	0.25
GA	69 (20.9%)	261 (79.1%)		
CSEA	42 (17.1%)	204 (82.9%)		
Waiting time (d)	5 (4-7)	5 (4-7)	1.83	0.70
Hypertension			1.69	0.19
No	70 (17.8%)	323 (82.2%)		
Yes	41 (22.4%)	142 (77.6%)		
Diabetes			5.08	0.02*
No	85 (17.6%)	397 (82.4%)		
Yes	26 (27.7%)	68 (72.3%)		
Smoking			5.08	0.02*
No	99 (21.0%)	372 (79%)		
Yes	12 (11.4%)	93 (88.6%)		
Alcohol			2.93	0.09
No	96 (20.6%)	369 (79.4%)		
Yes	15 (13.5%)	96 (86.5%)		
Surgical approach			0.09	0.75
HA	87 (19.6%)	358 (80.4%)		
IF	24 (18.3%)	107 (81.7%)		
Type of fracture			3.63	0.06
FNF	87 (21.3%)	322 (78.7%)		
ITF	24 (14.4%)	143 (85.6%)		

*Abbreviations*: *BMI* body mass index, *Hb* hemoglobin, *TXA* tranexamic acid, *GA* general anesthesia, *CSEA* combined spinal and epidural anesthesia, *HA* hip arthroplasty, *IF*, internal fixation, *FNF* femoral neck fracture, *ITF* inter-trochanteric fracture

*: Statistically significant difference

**Table 2:** Univariate and multivariate logistic analysis of risk factors for blood transfusion after hip fracture surgery

	Univariate		Multivariate	
	*OR* (*95% CI*)	*P*	*OR* (*95% CI*)	*P*
Age (year)	1.07 (1.044-1.09)	< 0.01*	1.03 (1.01-1.06)	0.02*
Sex				
Female	Ref.		Ref.	
Male	0.53 (0.33-0.84)	0.01*	1.08 (0.56-2.10)	0.82
BMI (kg/m^2^)	0.90 (0.84-0.96)	< 0.01*	0.92 (0.85-1.01)	0.06
Hb (g/L)	0.92 (0.90-0.93)	0.01*	0.92 (0.90-0.94)	< 0.01*
TXA				
No	2.28 (1.45-3.57)	< 0.01*	1.92 (1.11-3.34)	0.02*
Yes	Ref.		Ref.	
Type of anesthesia				
GA	1.28 (0.84-1.96)	0.25	2.08 (1.21-3.57)	< 0.01*
CSEA	Ref.		Ref.	
Waiting time (d)	1.11 (1.01-1.21)	0.04*	0.93 (0.82-1.04)	0.19
Hypertension				
No	Ref.		Ref.	
Yes	1.33 (0.86-2.05)	0.19	1.14 (0.66-1.96)	0.65
Diabetes				
No	Ref.		Ref.	
Yes	1.79 (1.07-2.97)	0.03*	1.55 (0.83-2.92)	0.17
Smoking				
No	Ref.		Ref.	
Yes	0.49 (0.26-0.92)	0.03*	0.55 (0.22-1.41)	0.22
Alcohol				
No	Ref.		Ref.	
Yes	0.60 (0.33-1.08)	0.09	1.10 (0.45-2.73)	0.83
Surgical approach				
HA	Ref.		Ref.	
IF	0.92 (0.56-1.52)	0.75	1.62 (0.45-5.78)	0.46
Type of fracture				
FNF	Ref.		Ref.	
ITF	0.62 (0.38-1.02)	0.06	0.51 (0.14-1.85)	0.30

Abbreviations: *BMI* body mass index, *Hb* hemoglobin, *TXA* tranexamic acid, *GA* general anesthesia, *CSEA* combined spinal and epidural anesthesia, *HA* hip arthroplasty, *IF* internal fixation, *FNF* femoral neck fracture, *ITF* inter-trochanteric fracture

*: Statistically significant difference

**Fig. 2:**
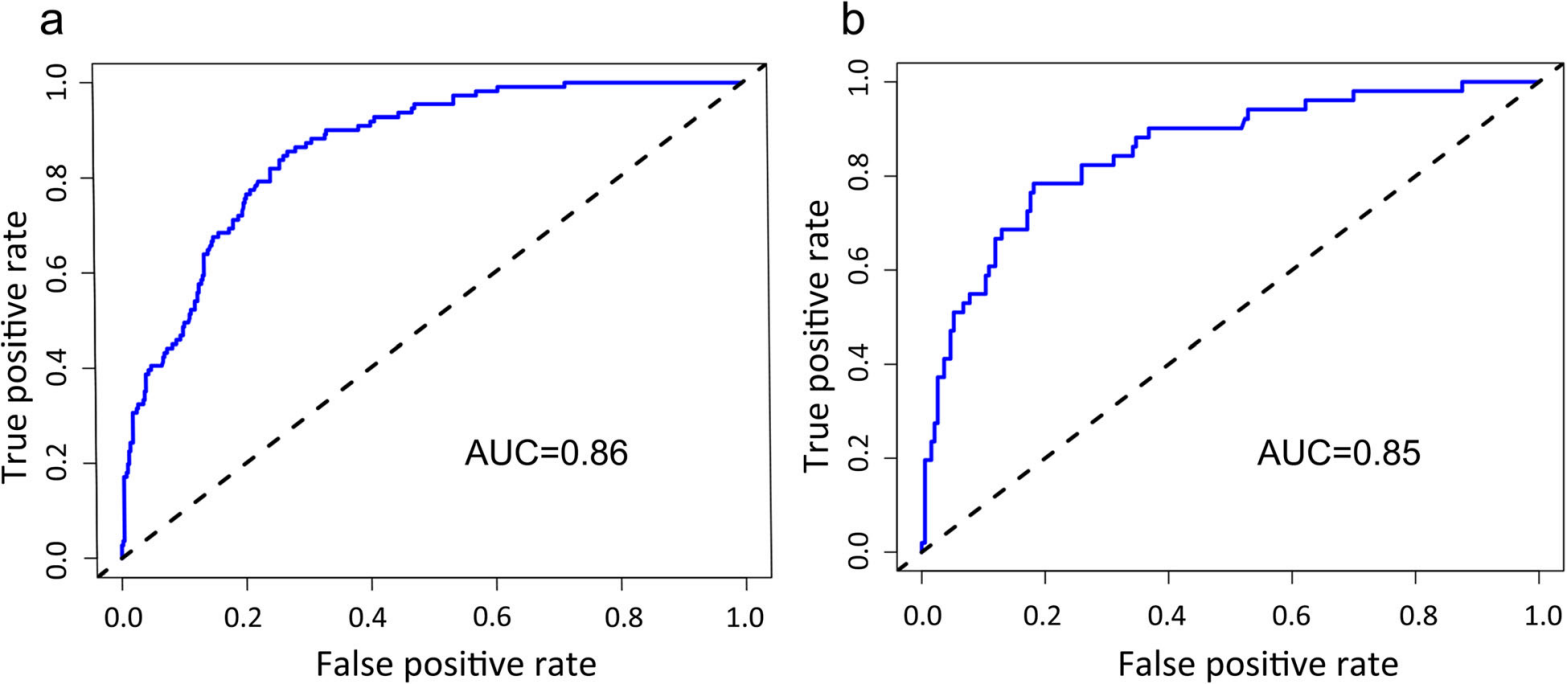
Comparison of the area under the receiver operating characteristic curve between nomogram-independent predictors in the training set (**a**) and the testing set (**b**)

**Fig. 3:**
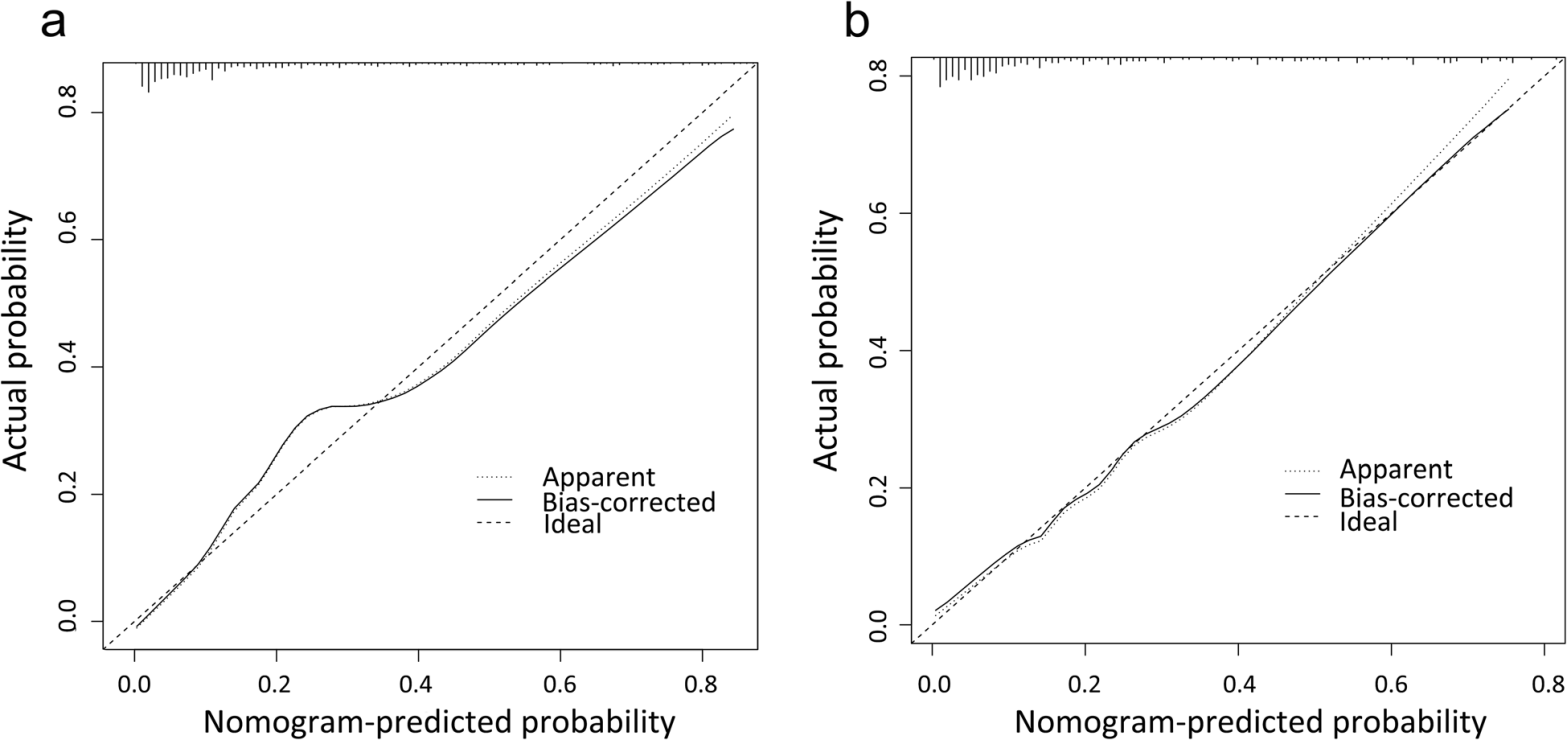
Comparison of calibration curves between the training set (**a**) and the testing set (**b**)

**Fig. 4:**
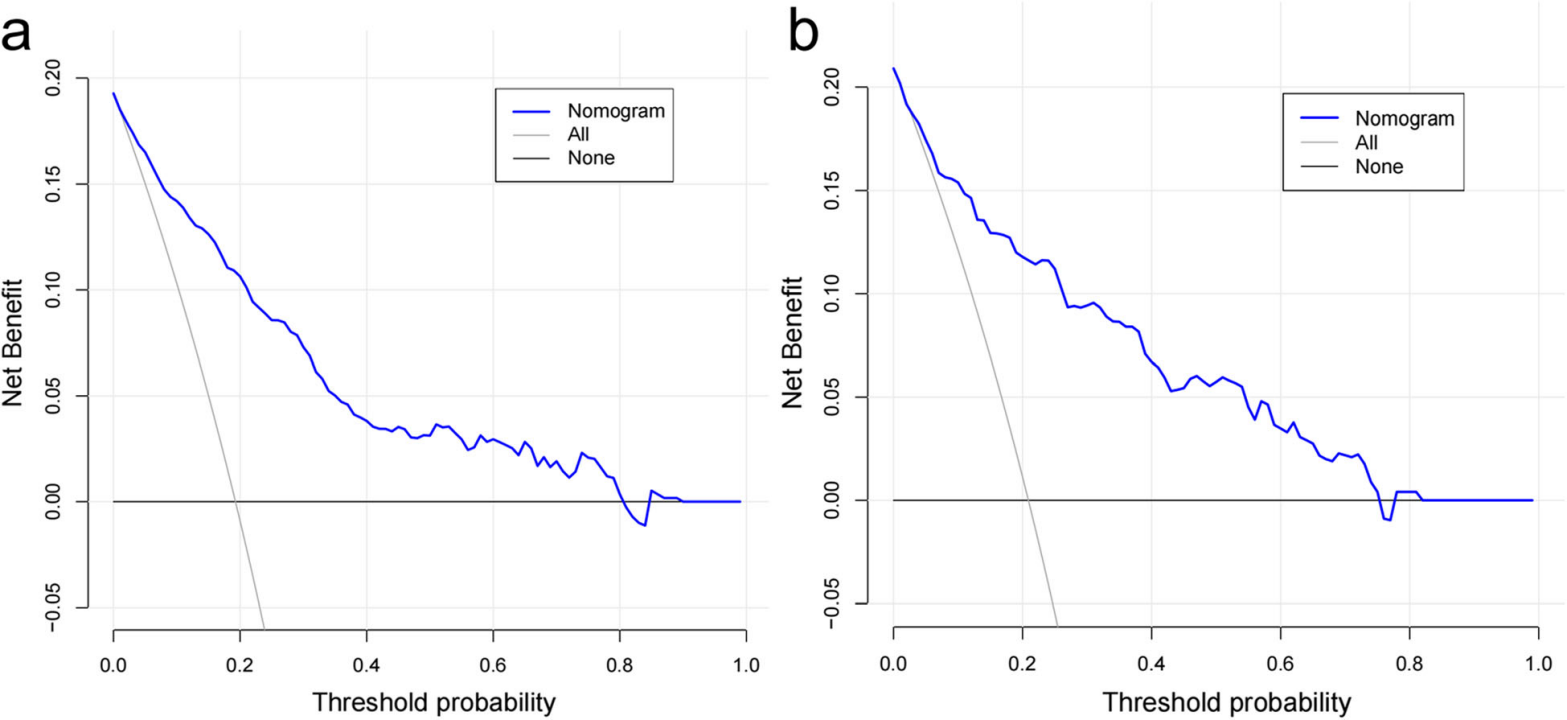
Comparison of decision curve analyses between the training set (**a**) and the testing set (**b**)

The accuracy of the nomogram model was estimated by internal validation in the training set and testing sets. This model’s C-index was 0.86 (95% CI, 0.83~0.89), which indicated that the model was predictive with high accuracy. Furthermore, the ROC curve was constructed, and the AUC was calculated for both the training and testing sets. The AUC was 0.86 in the training set (Fig. 2a) and 0.85 in the testing set (Fig. 2b), illustrating that the model had high discrimination. The calibration curves demonstrated good consistency between the model’s actually observed probability and the predicted probability (Fig. 3). DCA indicated that this nomogram model could be an excellent prediction tool for blood transfusion after hip fracture operation (Fig. 4).

The use of the nomogram is straightforward. The total score of prognostic indicators can be obtained by adding the individual scores, and then the total score can be used to calculate the probability of blood transfusion. For example, a 70-year-old patient whose preoperative hemoglobin was 100 g/L decided to use general anesthesia during the operation and did not use TXA during the perioperative period. The patient’s age score was 25, the preoperative Hb score was 75, the general anesthesia score was 11, and the not used TXA score was 8. The total score was 25 + 75 + 11 + 8 = 119, which was equivalent to 60% of the risk of blood transfusion.

Postoperative patients received routine treatment. Antibiotics were used to prevent infection within 3 days after the operation; symptomatic support was given to patients with mild fever after blood transfusion; all mild skin infections were cured after debridement and dressing changes. Patients received low-molecular-weight heparin to prevent postoperative deep vein thrombosis 12 h after the operation and were changed to rivaroxaban after discharge to 1 month after the operation.

## Discussion

Among the complications of hip fracture, 40-80% of patients show symptoms of anemia, which is considered one of the threatening complications in hip fracture [14], and correcting anemia is beneficial to improve the patient prognosis. However, related studies have pointed out that perioperative blood transfusion cannot reduce the postoperative mortality of hip fracture but will further increase the postoperative infection risk [15]. Therefore, reducing the risk of blood transfusion is a problem for all surgeons. Therefore, constructing a nomogram in this study can make it more intuitive to evaluate a patient’s probability of postoperative blood transfusion.

Using the nomogram model, preoperative risk factors can be assessed at an early stage, and surgeons can apply interventions to reduce the waste of blood products. This study concluded that low preoperative Hb was one of the independent risk factors for postoperative blood transfusion, which is consistent with previous studies [9, 16]. The preoperative Hb of the non-transfused group was 126.62 ± 15.18, which was significantly higher than that of the transfused group (105.96 ± 14.08) (P < 0.05). Hip surgery mainly involved GA or CSEA, and the univariate results showed that the type of anesthesia was not statistically significant. In contrast, the multivariate analysis showed that the type of anesthesia was statistically significant, and GA was 2.08 (95% CI, 1.21-3.57). First, CSEA may decrease intraoperative bleeding by keeping patients hypotensive intraoperatively through reducing the pressure on the arteriovenous system [17]. Second, the inhaled anesthetic mixture during GA hinders the formation of erythrocytes during the recovery of erythrocyte endogenesis, which eventually leads to the aggravation of anemia in patients [18]. Previous studies have also reported similar results [9]. Therefore, CSEA can reduce intraoperative bleeding and improve surgical safety.

TXA is widely used in clinical work, especially in orthopedic fields involving the joints and the spine, thereby reducing the perioperative bleeding risk [19]. Evidence shows that the safety and effectiveness of tranexamic acid play an important role in the fast-track procedures [20]. TXA stabilizes fibrin clots by binding plasmin to fibrin to inhibit plasminogen activation, resulting in more stable hemostasis [21]. Yang et al. [22] confirmed that after the application of TXA, the blood transfusion rate of hip replacement decreased from 22.4 to 5.7%. Research has shown that patients who receive TXA have significantly less total blood loss than patients who do not [23].

Among the patients who were used to develop the model, those older than 65 years who received blood transfusion accounted for 27.4% of the elderly patients, and those younger than 65 years who received a transfusion accounted for 6.3% of their age group. There was a significant difference between the two groups. Some authors similarly consider older age of hip fracture patients as a risk factor for postoperative blood transfusion [16, 24, 25]. Gruson et al. [26] suggested that each 5-year increase in age is associated with a 32% relative increase in the risk of transfusion, with an almost threefold increase in the risk of transfusion among patients older than 65. Elderly patients are prone to develop decreased hematopoietic activity, decreased platelet function, and a lower transfusion threshold by physicians [27]. Therefore, advanced age is an independent risk factor for postoperative blood transfusion. There is no consensus on whether sex is an independent risk factor for postoperative transfusion. Some authors have suggested that female patients have a higher risk of postoperative transfusion than male patients [26]. But in the present study, the results were shown not to be independent risk factors by binary regression analysis, in keeping with previous findings [12].

The present study has some shortcomings: (1) It was adopted to be a retrospective study. The data were from a single center, there was no multicenter analysis, and the discriminatory ability of this model in prediction needs to be confirmed by external tests. (2) The study period was relatively long, and there may be differences regarding surgical techniques and surgical medication; therefore, a cross-sectional study should be conducted.

## Conclusions

This study found that risk factors affecting postoperative blood transfusion were highly correlated with preoperative Hb, type of anesthesia, TXA, and age. This study developed a nomogram model; the advantages of which can transform complex regression equations into a visual and straightforward graph, make the results of the prediction model readable, and have a higher use value. Our established nomogram enables a relatively accurate assessment of the risk of postoperative blood transfusion in hip fracture, guides the surgeon to preoperative interventional treatment, and reduces the rate of postoperative blood transfusion.

## Data Availability

The datasets used and/or analyzed during the current study are available from the corresponding author on reasonable request.

## Abbreviations

C-index: Concordance index
ROC curve: Receiver operating characteristic curve
DCA: Decision curve analysis
Hb: Hemoglobin
GA: General anesthesia
CSEA: Combined spinal-epidural anesthesia
TXA: Tranexamic acid
BMI: Body mass index
HA: Hip arthroplasty
IF: Internal fixation
FNF: Femoral neck fracture
ITF: Inter-trochanteric fracture
